# Biological potency and characterization of antibacterial substances produced by *Lactobacillus pentosus* isolated from *Hentak*, a fermented fish product of North-East India

**DOI:** 10.1186/s40064-016-3452-2

**Published:** 2016-10-07

**Authors:** Chirom Aarti, Ameer Khusro, Mariadhas Valan Arasu, Paul Agastian, Naïf Abdullah Al-Dhabi

**Affiliations:** 1 Research Department of Plant Biology and Biotechnology, Loyola College, Nungambakkam, Chennai, Tamil Nadu 600034 India; 2 Department of Botany and Microbiology, Addiriyah Chair for Environmental Studies, College of Science, King Saud University, P. O. Box 2455, Riyadh, 11451 Saudi Arabia

**Keywords:** Antagonistic substances, Cell free neutralized supernatant, *Hentak*, Lactic acid bacteria, OFAT, *Lactobacillus pentosus*

## Abstract

Lactic acid bacteria (LAB) isolated from various foods are important due to their potential to inhibit microorganisms, including drug-resistant bacteria. The objectives of this investigation were to isolate and identify antibacterial substances producing LAB from *Hentak*, a traditional fermented fish product of Manipur (North-East India), and to optimize the production of antagonistic substances present in cell free neutralized supernatant (CFNS) against enteric bacterial pathogens using the ‘one factor at a time’ (OFAT) method. Out of 10 LAB, the most potent bacterium producing antibacterial substances was isolated and identified as *Lactobacillus pentosus* strain LAP1 based upon morphological, biochemical and molecular characterization. MRS (de Man, Ragosa and Sharpe) medium was determined to provide better bactericidal activity (AU/ml) than other tested media against the indicator enteric bacteria, including *Staphylococcus epidermidis* MTTC 3615, *Micrococcus luteus* MTCC 106, *Shigella flexneri* MTCC 1457, *Yersinia enterocolitica* MTCC 840 and *Proteus vulgaris* MTCC 1771. The culture conditions (pH: 5, temperature: 30 °C and inoculum volume: 1 %) and medium components (carbon source: lactose and nitrogen source: ammonium chloride) were observed to be the most influential parameters of significant antagonistic activity of CFNS against the enteric pathogens. MRS medium supplemented with Tween20 effectively stimulated the yield of antibacterial substances. The CFNS of strain LAP1 exhibited sensitivity to proteolytic enzyme (pepsin) treatment and heat treatment (60 °C for 60 min, 100 °C for 30 min and 121 °C for 15 min) and lost its inhibitory properties. The CFNS was active at an acidic (pH 3.0) to neutral pH (pH 7.0) but lost its antagonistic properties at an alkaline pH. The CFNS obtained from strain LAP1 scavenges the DPPH (1,1-diphenyl-2 picrylhydrazyl) significantly in a concentration-dependent manner within the range of 8.8 ± 0.12–57.35 ± 0.1 %. The OFAT-based approach revealed the baseline for statistical optimization, the scale-up process and efficient production of CFNS by *L. pentosus* strain LAP1, which could be used as a potential antibacterial and free radical scavenging agent.

## Background

Fermented foods are among the essential constituents of the human diet. Fermented food products are considered a good source of industrially important microorganisms (Rejiniemon et al. 2015; Jagadeesh 2015; Ilavenil et al. 2015). Similar to other states in North-East India, Manipur has a rich tradition in food processing and preservation technologies. Fermented foods of aquatic origin are still widely prepared and consumed in Manipur. *Hentak* is a highly consumed fermented fish product in Manipur and is mainly prepared at the household level in a cost-effective manner. However, there is a lack of knowledge regarding the bacteria involved in the increased shelf life of these products and the health benefits of those bacteria in humans. Therefore, an attempt had been made to isolate bacteria that produce antibacterial substances from *Hentak* and to characterize their inhibitory activity against human enteric pathogens.

Probiotics are non-pathogenic, known to compete with pathogens for available space by secreting lytic enzymes, organic acids and bacteriocins, inhibiting the growth of pathogens by disrupting their virulent gene expression, attachment and cell to cell communication, although widely adopted, is not acceptable to the European Food Safety Authority because it embeds a health claim which is not measurable (Pena et al. 2007; Ravi et al. 2007; Verschuere et al. 2000). Lactic acid bacteria (LAB) or probiotics from fermented foods are major resources for antimicrobial biosynthesis. Gram positive and non-sporulating bacteria play a prominent role in the production of growth inhibitory substances. LAB are safe and play an important role in food fermentation and preservation. The genus Lactobacillus belongs to the lactic acid bacteria that are rod shaped, Gram positive and non-spore forming. *Lactobacillus pentosus* is a lactic acid bacterium commonly used as starter culture for the fermentation process (Ruiz-Barba et al. 1994). Certain strains of *L. pentosus* exert probiotic properties, improve mucosal immunity and create resistance towards bacterial infections (Kotani et al. 2010; Izumo et al. 2011).

Environmental factors such as pH, temperature and medium composition can influence the production of antagonistic substances from lactic acid bacteria. Several reports have been discussed regarding antibacterial components, especially bacteriocin production by LAB and its optimizing by altering several physical factors and medium composition (Parente et al. 1994; Moortvedt-Abildgaard et al. 1995; De Vuyst and Vandamme 1992). Lactic acid bacteria and their specific components could be an eco-friendly antibacterial substitute for synthetic antibiotics. There is a continuous effort by worldwide researchers to optimize culture conditions and other parameters for the efficient production of antibacterial components from LAB that mitigate the growth of human pathogens. Therefore, in light of the over demand of antibacterial substances for therapeutic applications, the present study had been undertaken to investigate the influence of various culture conditions and medium components on the production of CFNS by a ‘one factor at a time’ (OFAT)-based approach using lactic acid bacteria isolated from *Hentak*.

## Methods

### Sample preparation

Fresh water small fish, *Ngasang* (*Esomus danricus*), were smoked and sun dried until they crumbled. The petioles of an aroid plant, *Khonagu* (*Alocasia macrorhiza*) were cut into small pieces, washed with water and sun dried for an hour. The crumbled fish powder was crushed with plant material in a 1:1 ratio using a stone mortar and pestle to make a paste. The mixture was kneaded with clean hands to produce ball-shaped pieces, and fermentation was allowed by keeping the mixture at room temperature for 5–6 days in an earthen pot containing a thin layer of banana leaves. The ball-shaped pieces were taken out from the pot and mixed with onions and mustard oil. The mixture was kneaded again using a stone mortar and pestle and made into a ball shape. The ball-shaped pieces were kept again inside the earthen pot containing banana leaves for 2–3 days. The fermented non-salted fish product, *Hentak*, was brought to the laboratory for the bacterial isolation process. The involvement of fish in the experiments was approved by the Government of India Ethical Committee (IAEC-LC 05/13).

### Isolation of lactic acid bacteria

One gram of *Hentak* was ground with sterilized distilled water using a mortar and pestle cleaned with ethanol (95 % w/v). The mixture was centrifuged at 8000×*g* for 15 min in order to remove heavy particles, and the supernatant was collected. The supernatant was serially diluted (10^−1^–10^−5^) for bacterial enumeration, and 1 ml of the suspension was poured onto sterilized MRS agar (g/l—proteose peptone 10.0, beef extract 10.0, yeast extract 5.0, dextrose 20.0, polysorbate 80 1.0, ammonium citrate 2.0, sodium acetate 5.0, magnesium sulfate 0.1, manganese sulfate 0.05, dipotassium phosphate 2.0, pH 6.5, Agar 18) plates. After spreading the suspension, the plates were incubated at 30 °C for 48 h. The total number of viable colonies was counted and expressed as colony forming units (CFU/ml). Based upon morphology, various colonies were selected for the isolation of pure bacterial cultures on MRS agar slants.

### Bacteria of interest

Indicator bacterial strains (human enteric pathogens) were collected from the Department of Plant Biology and Biotechnology, Loyola College, Chennai, India. Both Gram positive (*Staphylococcus epidermis* MTTC 3615, *Staphylococcus aureus* MTCC 96, *Enterococcus faecalis* MTCC 439 and *Micrococcus luteus* MTCC 106) and gram negative (*Shigella flexneri* MTCC 1457, *Yersinia enterocolitica* MTCC 840, *Enterobacter aerogens* MTCC 111 and *Proteus vulgaris* MTCC 1771) bacteria were used for the present study. The indicator bacterial cultures were sub-cultured selectively onto basal media (nutrient broth for gram positive and Mueller–Hinton broth for gram negative bacteria, pH 7.0) at 37 °C for further study. A 24 h old bacterial culture was used for further experiments.

### Assay for antibacterial substance production

The isolated lactic acid bacteria were screened individually for the production of antagonistic substances. The lactic acid bacteria were inoculated individually into sterilized MRS broth and incubated for 48 h at 30 °C. The indicator microorganisms were inoculated into Nutrient broth and Mueller–Hinton broth for 24 h at 37 °C and swabbed onto Mueller–Hinton agar (MHA) plates. Agar plates were punched using a sterilized, flamed and alcohol-dipped cork borer, and 5 mm wells were created. The lactic acid bacteria were centrifuged at 8000×*g* for 10 min, and the culture supernatant was subjected to membrane filtration (0.22 µm). The sterilized cell free supernatant was neutralized (pH 7.0) using 1 N NaOH in order to exclude the antibacterial effect of organic acids in the medium. The cell free neutralized supernatant (CFNS) was treated individually with catalase (Sigma, India; 1 mg/ml) and incubated at 37 °C for 2 h in order to eliminate the inhibitory effect of hydrogen peroxide. After catalase treatment, the CFNS obtained from lactic acid bacteria was then assayed for antibacterial assay against indicator bacteria using the agar well diffusion method. The growth inhibitory activity was expressed in arbitrary units (AU/ml). One AU was defined as the reciprocal of the highest level of dilution resulting in a clear zone of growth inhibition (Bhaskar et al. 2007).

### Identification and molecular characterization of the isolate

The potent bacterium was identified using morphological and biochemical tests and further characterized using molecular tools. The genomic DNA of the potential isolate was isolated and purified using a QIAquick^®^ kit (Qiagen Ltd., Crawley, UK). The amplicon sequencing was performed using universal primers 27F (5′ AGA GTT TGA TCG TGG CTC AG 3′) and 1492R (3′ GCT TAC CTT GTT ACG ACT T 5′). The 16S rRNA sequence of the isolate was subjected to BLAST, NCBI. Then, the sequence of the isolate was deposited into NCBI Genebank, and an accession number was assigned. The potential isolate was used for further experiments.

### Media optimization

*Lactobacillus pentosus* strain LAP1 was inoculated individually into 250 ml conical flasks containing sterile production medium (50 ml) such as Nutrient broth, Mueller–Hinton broth, Luria–Bertani broth, MRS broth and Peptone broth to compare the production of antibacterial substances. The flasks were incubated at 30 °C for 48 h in an orbital shaker (120 rpm). The CFNS was obtained, and the arbitrary units (AU/ml) were estimated as described above against the indicator bacteria.

### Optimization of culture conditions and medium components using the OFAT method

The suitable production media was optimized using various culture conditions (pH, temperature and inoculum volume) and medium components (carbon sources and nitrogen sources) utilizing the OFAT method after working out a series of experiments. The fermentation conditions and medium components were substituted one by one by keeping other factors constant in the production medium. The antibacterial substance production by strain LAP1 was examined by adjusting the pH (4, 5, 6, 7 and 8) of the production medium using 1 N HCl and 1 N NaOH. Similarly, the production of antagonistic substances with strain LAP1 was optimized by varying their respective conditions such as incubation temperature (20–70 °C) and inoculum volume (0.5–2 %) at the optimized pH. Likewise, the various media components such as carbon sources (maltose, fructose, sucrose, lactose, and xylose individually at 1.0 % w/v) and nitrogen sources (ammonium acetate, ammonium chloride, ammonium nitrate, ammonium sulphate and sodium nitrate individually at 0.5 % w/v) were substituted in the production medium in order to achieve maximum production of antibacterial substances. An appropriate control medium was also maintained. All of the flasks were aseptically inoculated with the isolate and kept in an orbital shaker (120 rpm) for 48 h. The CFNS was collected after centrifugation at 8000×*g* for 10 min, followed by membrane (0.22 μm) filtration of the supernatant and neutralization. The CFNS was collected and the antibacterial activity (AU/ml) was examined against the most susceptible indicator bacteria as described above.

### Effect of supplements on antibacterial substance production

The production of antibacterial substances by *L. pentosus* strain LAP1 was assessed under optimized culture conditions in the suitable production medium supplemented with Tween20 (1 % v/v), Tween40 (1 % v/v) Tween80 (1 % v/v) and glycerol (1 % v/v). An appropriate control medium was also maintained, and the antagonistic activity of CFNS was determined as described above using the most susceptible indicator organisms.

### Characterization of CFNS

The CFNS from strain LAP1 was characterized with respect to pH, heat treatment and proteolytic enzymes. The stability of CFNS at different pH values (pH 3, 5, 7, 8 and 10) was tested by adjusting the pH of the supernatant with either 1 N HCl or 1 N NaOH. The adjusted supernatants were incubated for 4 h at room temperature, and the activity was calculated using indicator bacteria. The CFNS of the isolate was subjected to heat treatment at temperatures of 60 °C for 60 min, 100 °C for 30 min, and autoclaving (121 °C/15 min). CFNS and H_2_O_2_-eliminated CFS (cell-free supernatant) without any heat treatment served as a control. Aliquots of each treatment were taken after the required incubation period, and the activity of heat treated CFNS was determined against indicator bacteria as described earlier using the agar well diffusion method. Similarly, the sensitivity of inhibitory substances produced by the isolate to proteolytic enzyme such as pepsin (1 mg/ml) was determined. The reaction mixtures were then incubated at 37 °C for 1 h, and the antagonistic activity of the supernatant was determined as described above.

### Determination of DPPH free radical scavenging activity

The DPPH (2,2-diphenyl-1-picrylhydrazyl) assay is one of the most commonly used methods to detect free radical scavenging activity. The DPPH scavenging assay for the CFNS of strain LAP1 was measured by the method of Chen et al. (2005) with some modifications. Various concentrations (100–1000 µl) of CFNS were mixed with 1 ml of 0.05 mM DPPH solution. The reaction was incubated in the dark at room temperature for 30 min. DPPH solution was used as a control, and a combination of CFNS and methanol was used as the blank. The DPPH scavenging capacity of the CFNS of the isolate was calculated by measuring the decrease in absorbance at 517 nm compared to the control. The DPPH scavenging capacity was calculated as:

$${\text{DPPH scavenging capacity (\% )}} = \left[ {{{\left( {{\text{A}}_{{{\text{sample}}}}  - {\text{A}}_{{{\text{blank}}}} } \right)} \mathord{\left/ {\vphantom {{\left( {{\text{A}}_{{{\text{sample}}}}  - {\text{A}}_{{{\text{blank}}}} } \right)} {{\text{A}}_{{{\text{control}}}} }}} \right. \kern-\nulldelimiterspace} {{\text{A}}_{{{\text{control}}}} }}} \right] \times 100$$

### Statistical analysis

All of the experiments were performed in triplicate, and the data were calculated as the Mean ± SD with MS-Excel.

## Results

### Isolation of lactic acid bacteria

Countable colonies of lactic acid bacteria were observed from dilutions of 10^−3^–10^−5^. The lactic acid bacteria from *Hentak* ranged from 209.0 ± 5.03 to 85.0 ± 4.0 CFU/mL at 10^−3^–10^−5^ dilutions, respectively. In the present study, 10 lactic acid bacteria were isolated on MRS agar plates based upon distinct morphologies (data not shown).

### Screening for antibacterial substance production

Screening for potential antagonistic activity of all the isolates against the indicator bacteria was performed using the agar well diffusion assay. Twenty percent of the isolates were found to be effective against most of the indicator bacteria. Based upon the diameter of the zone of inhibition shown by the catalase-treated CFNS of the most potent isolate, susceptible bacteria, including *Staphylococcus epidermidis, Micrococcus luteus, Shigella flexneri, Yersinia enterocolitica* and *Proteus vulgaris*, were selected for further optimization (data not shown).

### Identification and molecular characterization of the isolate

The most potent bacterium underwent morphological identification, biochemical property characterization and molecular characterization using 16S rRNA sequencing (data not shown). An amplicon of 1519 bp was observed using PCR amplification and sequencing. The sequence was subjected to a multiple sequence alignment using the BLAST analysis of NCBI. The 16S rRNA sequence showed a homology of 100 % with *L. pentosus*. The sequence was deposited in GenBank, maintained by NCBI, USA (Accession No: KU945826), and the organism was identified as *L. pentosus* strain LAP1.

### Media optimization

**Fig. 1:**
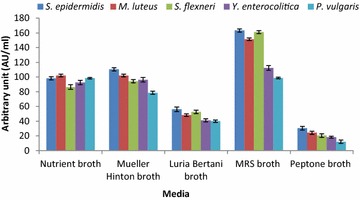
Effect of various media on the production of antibacterial substances by strain LAP1. MRS broth favours the increased production of antibacterial substances. Each point represents the mean ± standard error of three independent experiments

Strain LAP1 was cultured in various media in order to ensure the maximum production of antibacterial substances. MRS broth was found to be the most favourable medium for the maximal production of antagonistic substances (98.7 ± 1.1–163.3 ± 2.13 AU/ml). The other production media resulted in minimal yield of antibacterial constituents compared to MRS medium (Fig. 1). The antibacterial substance yield of various media was as follows: MRS broth > Mueller–Hinton broth > Nutrient broth > Luria–Bertani broth > Peptone broth. Peptone broth was found to be the least effective medium for antibacterial substance production from strain LAP1, ranging from 30.4 ± 2.33 to 12.1 ± 2.31 AU/ml against the most susceptible indicator bacteria.

### Optimization of culture conditions and medium components

**Fig. 2:**
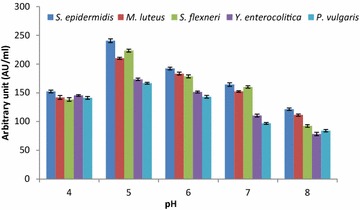
Effect of pH on the production of antibacterial substances by strain LAP1. An acidic pH (pH 5) favours the increased production of antibacterial substances from the isolate. Each point represents the mean ± standard error of three independent experiments

Subsequent investigation was carried out to optimize the production of antibacterial substances (AU/ml) from strain LAP1 using the OFAT method. The culture conditions, such as pH and temperature, were optimized for maximum production of growth inhibitory substances. The production of antibacterial components was enhanced by adjusting the pH of the MRS broth. Among the tested pH, the maximum production in terms of antagonistic activity was recorded at pH 5.0 and ranged from 166.6 ± 1.65 to 240.5 ± 3.18 AU/ml. However, a further decrease or increase of pH was found to mitigate the production of antibacterial substances significantly. The minimum production was recorded at pH 8.0 and ranged from 84.1 ± 2.08 to 121.4 ± 2.17 AU/ml against the control range (pH 7.0) of 96.7 ± 1.67 to 164.3 ± 3.08 AU/ml (Fig. 2).

**Fig. 3:**
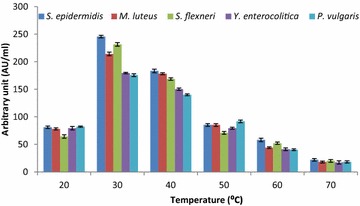
Effect of various incubation temperatures on the production of antibacterial substances by strain LAP1. The isolate showed enhanced antibacterial substance production at 30 °C. Each point represents the mean ± standard error of three independent experiments

Figure 3 shows the effect of incubation temperature on antibacterial substance production from strain LAP1. The maximum production of 175.6 ± 2.34 to 245.5 ± 2.41 AU/ml was recorded at 30 °C, and a temperature lower or higher than 30 °C markedly decreased the production of antibacterial substances. The minimum yield was within the range of 18.3 ± 2.08 to 22.1 ± 2.17 AU/ml at 70 °C over the control range.

**Fig. 4:**
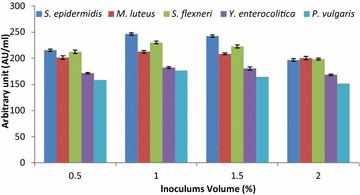
Effect of inoculum volume on the production of antibacterial substances by strain LAP1. Inoculum volumes lower than or greater than 1 % did not have a large effect on antibacterial substance production. Each point represents the mean ± standard error of three independent experiments

Different inoculums of strain LAP1 did not show any significant effect on the antagonistic activity of CFNS obtained against the indicator enteric bacteria (Fig. 4). The antibacterial substance production was higher (168.4 ± 2.41 to 305.4 ± 2.43 AU/ml) at the 1 % inoculum level. However, no further increase in production was observed at lower (0.5 %) or higher volumes of inoculum (2 %).

**Fig. 5:**
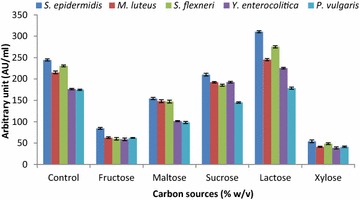
Effect of various substrates as carbon sources (% w/v) on the production of antibacterial substances by strain LAP1. By substituting for dextrose (original carbon source of MRS media, Control), lactose favours the maximum production of antibacterial substances from the isolate. Each point represents the mean ± standard error of three independent experiments

**Fig. 6:**
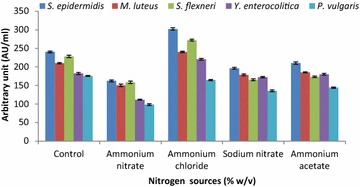
Effect of various nitrogen sources (% w/v) on the production of antibacterial substances by strain LAP1. Ammonium chloride addition increased antibacterial substance production when substituted for ammonium citrate (control). Each point represents the mean ± standard error of three independent experiments

Strain LAP1 produced growth inhibitory components at a higher level (178.3 ± 2.41–310.4 ± 2.43 AU/ml) when the carbon source of MRS medium was substituted with lactose. On the other hand, the minimum antagonistic activity (41.3 ± 1.67–54.2 ± 3.08 AU/ml) was observed in xylose supplied medium over the control MRS medium ranging from 174.6 ± 1.23 to 244.5 ± 2.43 AU/ml (Fig. 5). Similar to the carbon source, the nitrogen source also favoured the optimal production of antagonistic substances from strain LAP1 (Fig. 6). The production of antibacterial substances from the isolate was higher (164.3 ± 1.65–302.3 ± 3.18 AU/ml) in the presence of ammonium chloride. However, the minimum production (98.3 ± 2.34–162.3 ± 2.41 AU/ml) was obtained in ammonium nitrate supplied medium over the control range (175.6 ± 1.1–240.5 ± 2.13 AU/ml).

### Effect of supplements

**Fig. 7:**
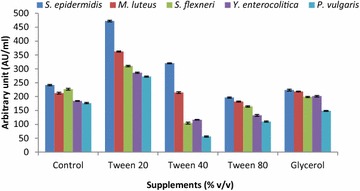
Effect of various supplements (% v/v) on the production of antibacterial substances by strain LAP1. MRS medium supplemented with Tween20 resulted in enhanced antibacterial substance production compared to control (MRS medium without any supplements). Each point represents the mean ± standard error of three independent experiments

MRS medium supplemented with Tween20, Tween40, Tween80 and glycerol markedly affected the production of antibacterial substances by the candidate bacterium. The largest amount of antibacterial components (272.2 ± 1.65–472.3 ± 3.18 AU/ml) was produced in the MRS medium supplemented with Tween20 compared to the other tested supplements. Incorporation of Tween40, Tween80 and glycerol decreased the antagonistic activity of CFNS compared to the control range (Fig. 7).

### Characterization of the CFNS of strain LAP1

The stability of the catalase-treated CFNS of strain LAP1 at different pH and temperatures and in the presence of proteolytic enzymes is presented in Table 1. The antibacterial substances showed activity (AU/ml) at pH 3, 5 and 7 (control). However, elevating the pH toward alkaline conditions diminishes the antagonistic activity of the CFNS against the indicator bacteria. The CFNS of the isolated strain did not show any antagonistic activity against the indicator bacteria at pH >7.0. Heating the CFNS of strain LAP1 at 60 °C for 60 min, 100 °C for 30 min, and 121 °C for 15 min completely abolished the antagonistic activity of the bacteriocin against all of the indicator bacteria tested. Likewise, all of the potential proteinaceous components present in the CFNS of strain LAP1 were completely inactivated by the pepsin, resulting in the disappearance of the zone of inhibition on the agar plates inoculated with indicator bacteria.

**Table 1:** Characterization of bacteriocin from *Lactobacillus pentosus* strain LAP1 against most susceptible indicator bacteria

Factors	Indicator bacteria				
	*S. epidermidis*	*M. luteus*	*S. flexneri*	*Y. enterocolitica*	*P. vulgaris*
*pH stability*					
3	+	+	+	+	+
5	+	+	+	+	+
7 (control)	+	+	+	+	+
8	−	−	−	−	−
10	−	−	−	−	−
*Thermal stability*					
Control (unheated)	+	+	+	+	+
60 °C (60 min)	−	−	−	−	−
100 °C (30 min)	−	−	−	−	−
121 °C (15 min)	−	−	−	−	−
*Proteolytic enzyme*					
Control (untreated)	+	+	+	+	+
Pepsin treated	−	−	−	−	−

*+* zone of inhibition; *−* no zone of inhibition

### DPPH free radical scavenging activity

**Fig. 8:**
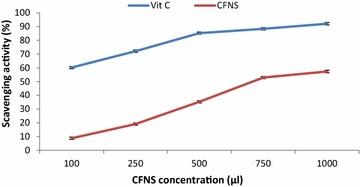
Effect of various concentrations (100–1000 µl) of the CFNS of strain LAP1 on DPPH scavenging activity. The results were compared with the free radical scavenging potential of the control (vitamin C). Each point represents the mean ± standard error of three independent experiments

Figure 8 shows the antioxidant activity of the CFNS of the isolate using DPPH free radicals. The scavenging potential of the cell free neutralized supernatant of the isolate increased significantly in a dose dependent manner (100–1000 µl). The antibacterial substance showed DPPH scavenging activity in the range of 8.8 ± 0.12–57.35 ± 0.1 % when compared to ascorbic acid (60.2 ± 0.11–92.1 ± 0.8 %).

## Discussion

The LAB produce a variety of antibacterial substances, including bacteriocins and bacteriocin-like components that inhibit the growth of pathogenic bacteria (Yasmeen et al. 2015; Ekhay et al. 2013). The isolation and screening of bacteria from natural sources is a successful way to obtain strains with valuable medical applications (Yang et al. 2012). All of the isolates of preliminary study in the present context revealed a varying degree of antagonistic activity against indicator organisms by secreting different types of antibacterial substances. The growth inhibition of indicator bacteria by catalase-treated CFNS provided evidence that the antagonistic activity might be due to the production of antibacterial components (Yasmeen et al. 2015). Previous studies had reported extensively on the dominance of LAB in fermented foods such as meat, fish, fruits, vegetables and dairy products (Grosu-Tudor et al. 2014; Hwanhlem et al. 2011).

Media play a very important role in the successful isolation of lactic acid bacteria and in maximizing the production of antibacterial substances from LAB. In the present context, strain LAP1 produced the maximum amount of inhibitory components in MRS medium. Our study favours earlier reports, which suggested that MRS medium was a better medium for the growth of probiotic bacteria and the production of antibacterial substances (Yang et al. 2012; Ten Brink et al. 1994). The low production of antibacterial substances recorded in other media suggests that the high yield of growth inhibitory components from the isolate depends upon the specific nutrients supplied in the medium for biomass production.

In the present investigation, the production of antibacterial substances from strain LAP1 was enhanced by optimizing the pH of the medium (pH 5.0). Our study strongly favours the findings of Iyapparaj et al. (2013), who demonstrated the maximum production of bacteriocin from lactic acid bacteria at pH 5.0. On the other hand, our results showed partial agreement with the findings of Zamfir et al. (2000), Aasen et al. (2000), Yang and Ray (1994) and Todorov and Dicks (2005), who observed maximum bacteriocin production in the range of pH 4.5-6.0. Maximum bacteriocin production was observed at an initial pH of 5.8, while a further increase in pH decreased the antagonistic activity (Verellen et al. 1998). In another similar study, a change in pH from lower to higher decreased the production of antibacterial substances in LAB (Cheigh et al. 2002). The variation in the production of growth inhibitory components with the change in the pH of the production medium might be due to changes in the biomass of the bacteria, post-translational modification or modification of the genes responsible for antagonistic characteristics (Liu and Chung 2005). In general, the production of antibacterial substances from strain LAP1 was stimulated at pH 5.0. Likewise, the effect of incubation temperature is a very critical parameter for the production of antibacterial substances such as bacteriocin (Delgado et al. 2007; Leaes 2011). Growth temperature and antagonistic substance production from lactic acid bacteria are often correlated, as indicated in the present report. Our study favours the findings of Iyapparaj et al. (2013) and Moonchai et al. (2005), who reported that the production of antibacterial substances from LAB was maximal at 30 °C. The present investigation was in complete agreement with the finding of Ekhay et al. (2013), who demonstrated that maximal antibacterial substance production by the bacterium correlates with the optimal cell growth temperature. However, the maximum production of growth inhibitory proteinaceous components was achieved at a temperature which was far from the incubation temperature required for cell growth (Messens and De Vuyst 2002). The inoculum volume (1 %) of strain LAP1 showed improved antagonistic activity of the CFNS against the indicator bacteria, but the rate of antibacterial substance production was not much influenced. This clearly indicated that the synthesis of growth inhibitory components from strain LAP1 was correlated with the specific cell biomass. Further extensive investigation is required to evaluate culture parameters to correlate the production of antibacterial substances and cell growth for specific strains.

In the present context, lactose was found to be the most effective sole substrate that favoured the enhancement of antibacterial component production from strain LAP1 towards the indicator bacteria. In agreement with our study, Iyapparaj et al. (2013), Abo-Amer (2011) and Moreno et al. (2003) showed maximum bacteriocin yield by LAB in the presence of lactose as a source of carbon in the production medium. On the other hand, the antagonistic activity of bacteriocin was increased when glucose was added to the medium (Ekhay et al. 2013; Todorov 2008). Previous reports and the present investigation clearly indicate that a specific substrate can induce or inhibit the antagonistic activity of the CFNS in a strain-dependent manner. According to the results obtained in our study, the rate of antibacterial substance production from strain LAP1 was affected by the addition of different nitrogen sources, but the antagonistic activity was not much influenced by the addition of ammonium chloride into the production medium. The investigation favours the finding of Ekhay et al. (2013), who demonstrated that the incorporation of inorganic nitrogen into the medium had no effect on the increased bacteriocin production. However, the present study was not in agreement with the finding of Iyapparaj et al. (2013), who reported that the increase in antagonistic activity was attributed to an inorganic nitrogen source, such as ammonium acetate. The production of antibacterial substances, such as bacteriocin, was also found to be inhibited due to the higher concentrations of nitrogen incorporated into the medium (Callewaert and De Vuyst 2000).

MRS medium supplemented with Tween20 induced the synthesis of antagonistic substances (Castro et al. 2011), as was also shown in the present study. The increased production by strain LAP1 in the presence of Tween20 in MRS broth might be because Tween20 is a non-ionic surfactant agent, and hence it has the ability to overproduce growth inhibitory components by affecting the bacterial cell membrane and secreting antibacterial substances directly into the medium. In other reports, broth supplemented with glucose and Tween80 inhibited the growth of indicator bacteria in a broad range by inducing the production of antibacterial substances (Iyapparaj et al. 2013; Verellen et al. 1998).

The CFNS showed stability and activity at both acidic and neutral pH (control). The results of the present study suggested that the antagonistic properties of the CFNS of the isolate against the indicator bacteria were due to the potent antibacterial substances, not because of the acidic environment. The stability of the antibacterial components at a low pH may be important in medicine as a potential antibacterial agent. These results are comparable with the reports of Messens and De Vuyst (2002), Yang et al. (2012) and Yasmeen et al. (2015), who demonstrated stability and better antagonistic activity of bacteriocins at an acidic pH.

Incubating the CFNS of strain LAP1 at different temperatures completely abolished the inhibitory properties of the antibacterial substances. These results demonstrated that the heat-labile antibacterial substances might be responsible for the inhibitory activity of the CFNS of the isolate. The results obtained in the current study provide one more significant step towards the study of the CFNS of *L. pentosus* as an antibacterial agent.

The sensitivity of the antibacterial substances towards proteolytic enzymes strongly established the proteinaceous nature of the CFNS obtained from *L. pentosus* strain LAP1. The result was in complete agreement with the findings of Bromberg et al. (2004), Sabia et al. (2014) and Yasmeen et al. (2015), who found that pepsin inhibited the antagonistic activity of most of the antibacterial substances produced by lactic acid bacterial strains.

Free radicals are the end product of metabolic process, and antioxidants are known to scavenge the free radicals produced inside the body. In the current study, the CFNS of strain LAP1 showed significant antioxidant properties (8.8 ± 0.12–57.35 ± 0.1 %) compared to ascorbic acid (60.2 ± 0.11–92.1 ± 0.21 %) in a concentration-dependent manner (100–1000 µl). Similar DPPH inhibition activity by LAB cell free supernatant was observed by Uugantsetseg and Batjargal (2014), who found that the antioxidant activity of the CFNS of isolates was in the range of 26.1–38.4 %. The DPPH free radical scavenging potential of the CFNS obtained from strain LAP1 may be involved in its antioxidant properties, and that is directly correlated with the concentration of antibacterial substances due to the presence of proteinaceous compounds and secondary metabolites in the CFNS of the isolate.

## Conclusion

From the present investigation, it is clear that *Lactobacillus pentosus* strain LAP1 isolated from *Hentak* produced antibacterial substances with growth inhibitory properties against human enteric pathogens. The maximum production of antibacterial substances was obtained in MRS broth supplemented with Tween20 utilizing optimized culture conditions and medium components. Additionally, the CFNS obtained from the isolate demonstrated antioxidant activity by scavenging DPPH in a dose-dependent manner. The OFAT optimization data on antibacterial substance production provides strong preliminary information for further investigation on the statistical optimization and bio-preservative role of CFNS for cost-effective industrial applications. The antagonistic substances from *L. pentosus* strain LAP1 could be used not only as a barrier to the growth of enteric pathogens but also for developing food products with antioxidant properties. An extensive study needs to be performed to explore the potency of antibacterial substances as an alternative therapy against disease-causing enteric bacteria.
